# Advanced screen-printed electrode functionalized ZnO/CNTs for the electrochemical analysis of opioid drug pethidine co administered with paracetamol: application in dosage form and human plasma sample

**DOI:** 10.1186/s13065-025-01599-8

**Published:** 2025-08-09

**Authors:** Shimaa A. Atty, Ahmed M. Abdelzaher, Sona Barghash, Mona A. Abdel Rahman

**Affiliations:** 1 Pharmaceutical Chemistry Department, Egyptian Drug Authority, Giza, 12561 Egypt; 2 Department of Pharmaceutical Analytical Chemistry, Faculty of Pharmacy, Al-Azhar University, Nasr City, Cairo, 11751 Egypt; 3 College of Pharmacy, University of Kut, Wasit, 52001 Iraq; 4 Analytical Chemistry Department, Faculty of Pharmacy, October 6 University, October 6 City, Giza, 12858 Egypt

**Keywords:** Screen printed electrode, ZnO, Multi-walled carbon nanotube, Pethidine, Paracetamol, Plasma

## Abstract

Synthetic opioids such as pethidine (PTD) have a high potential for addiction but are widely used in clinical settings to manage pain before and after surgical procedures. Combinations of pethidine (PTD) with paracetamol (PCM) are frequently prescribed for the treatment of severe and chronic pain. In this study, an eco-friendly voltammetric sensor based on a screen-printed electrode (SPE) modified with zinc oxide nanoparticles functionalized with carbon nanotubes (ZnO/CNTs) was developed and employed. For the first time, the proposed sensor was successfully applied for the determination of pethidine in pharmaceutical formulations, demonstrating excellent recovery. Furthermore, it enabled the simultaneous detection of pethidine and paracetamol in biological samples, with low detection limits of 980 pmol L⁻^1^ and 977 pmol L⁻^1^ for PTD and PCM, respectively. The sensor offers a promising tool for point of care diagnostics and routine analysis due to its sensitivity, simplicity, and cost-effectiveness.

**Supplementary Information:**

The online version contains supplementary material available at 10.1186/s13065-025-01599-8.

## Introduction

Surgical procedures are often associated with postoperative pain and complications, including increased morbidity [1]. Effective pain management is crucial to minimize these risks and support recovery [2]. Pethidine (PTD) (ethyl,1-methyl-4-phenylpiperidine-4-carboxylate hydrochloride), the first synthetic opioid of the phenylpiperidine class, acts as a µ-opioid receptor agonist and is widely used for postoperative pain relief [3]. Due to its high addiction potential, PTD is classified as a controlled substance and marketed as meperidine [4]. It has also been misused by athletes for pain relief and relaxation, often at doses exceeding medical guidelines, in attempts to boost performance [5].

Several studies have shown that pethidine (PTD) side effects are dose-dependent [6, 7]. To enhance analgesia and reduce opioid-related adverse effects, co-administration with nonsteroidal anti-inflammatory drugs (NSAIDs), such as paracetamol (PCM), is commonly recommended. This approach can either boost analgesic efficacy without increasing side effects or maintain effective pain control with lower opioid doses [8]. Paracetamol (N-(4-hydroxyphenyl) acetamide) [9], one of the most widely used NSAIDs [10], is valued for its analgesic and antipyretic properties. The combination of PTD and PCM is a standard clinical practice, offering effective pain relief with fewer side effects. Their synergistic interaction enhances analgesic efficacy compared to individual use, enabling reduced PTD dosages and minimizing risks such as respiratory depression and hypotension. Therefore, the simultaneous determination of both drugs is clinically significant.

Numerous analytical methods have been developed for the accurate determination of PTD, including tandem mass spectrometry and ultra-performance liquid chromatography (UHPLC-MS/MS) [11], high-performance liquid chromatography (HPLC) [12, 13], electro-chemiluminescence [14], gas chromatography ion trap mass spectrometry (GC–MS) [15], spectrophotometry [16, 17], and electrochemical and potentiometric techniques [18–24].

Compared to conventional techniques such as spectrophotometry, HPLC, and LC-MS, the proposed voltammetric method offers notable advantages for pethidine (PTD) analysis, including a lower limit of detection, higher sensitivity, and excellent selectivity. It enables direct analysis with minimal sample preparation, significantly reducing time and complexity. Additionally, the use of screen printed electrodes and low cost reagents makes the method economical and suitable for on-site or point of care applications, unlike the expensive and maintenance-intensive instrumentation required for chromatographic methods [25, 26].

Carbon based, nanomaterial enhanced electrochemical sensors especially screen printed electrodes (SPEs) have gained attention in pharmaceutical analysis due to their low cost, disposability, and ease of use [27–29]. Their adaptability allows for surface modification with nanomaterials to boost sensitivity and selectivity [30, 31]. In this study, we developed a novel ZnONPs/CNT-modified SPE (ZnONPs/CNT/MSPE) for PTD detection. Zinc oxide nanoparticles offer high stability, a large surface area, and excellent electrochemical properties [32, 33], while carbon nanotubes provide superior conductivity and enhanced electrode kinetics, enabling faster responses and lower detection limits [25]. Combining ZnO with CNTs improves particle morphology and overall sensor performance [34].

Compared to previously reported electrochemical methods, the proposed ZnO/CNT-modified screen-printed electrode (SPE) demonstrates significant improvements in analytical performance, notably achieving lower limits of detection (LOD), enhanced sensitivity, and excellent selectivity. Although ZnO/CNT composites have been explored in electrochemical sensing, their use for the simultaneous voltammetric determination of pethidine (PTD) and paracetamol (PCM) has not been previously reported.

Importantly, this study marks the first application of screen-printed electrodes for detecting the opioid drug PTD. We developed and optimized a novel ZnONPs/CNT-modified sensor (ZnONPs/CNT/MSPE) for rapid and sensitive detection of PTD, both individually and in combination with PCM. The sensor exhibited high selectivity for PTD, even in the presence of common interfering substances found in biological fluids and pharmaceutical formulations, such as uric acid, ascorbic acid, glucose, electrolytes, starch, sucrose, and cellulose. The electrochemical behavior, peak resolution, matrix compatibility, and detection parameters were thoroughly assessed and benchmarked against existing methods. These results highlight the analytical advantages and novelty of the proposed platform, particularly for clinical and pharmaceutical applications involving PTD and PCM co-administration.

## Materials and methods

### Instruments

All voltammetric measurements were performed using a Bio-Logic SP 150 electrochemical workstation. The screen-printed electrode (DropSens, DRP-110, Spain) comprised three electrodes: a Ag/AgCl reference electrode (in 100 mM KCl), a platinum wire counter electrode, and a working electrode.

Transmission electron microscopy (TEM) was conducted using a JEM-1400 electron microscope (Japan Electron Optics Laboratory), with samples positioned appropriately for analysis. Energy-dispersive X-ray spectroscopy (EDX) was performed using an FEI system (Netherlands) with an accelerating voltage of 30 kV, magnification ranging from 14× to 1,000,000, and a resolution of 1 nm.

pH measurements were carried out using a digital pH meter (Jenway Model 3505, Staffordshire, England) at ambient temperature. A Schmid Bauer sonicator (GmbH & Co. KG, Singen) and a Daihan magnetic stirrer (Model MSH-20 A) were used throughout the experimental procedures.

### Sample and reagents

#### Raw material and pharmaceutical dosage form

PTD (99.6% ± 0.1 purity) from Sigma-Aldrich in St. Louis, MO, USA, and PCM (99.88% ± 0.1 purity) from SIGMA Pharmaceutical Industries in Cairo, Egypt. The pethidine injection^®^, which the manufacturer claims contain 100 mg/2 mL per vial, was obtained from the local market. Plasma samples utilized in the study were collected from Vacsera Co. in Giza, Egypt.

#### Reagents and solvents

The support electrolyte was made by combining the Britton-Robinson (B-R) buffer with 0.04 mol/L acetic acid (Loba Chime Co., India), 0.04 mol/L phosphoric acid (Sigma-Aldrich), and 0.04 mol/L boric acid (El-Nasr Pharmaceutical Company, Le Caire, Egypt). The pHs of the buffer solutions were carefully adjusted by adding an appropriate volume of 0.2 M NaOH (Win lab, Leicestershire, U.K.). Graphite powder, paraffin oil, and CNTs were purchased from Sigma-Aldrich (Steinem, Germany). Normal solutions and buffers were prepared with Milli-Qultra-pure water (Millipore, Bradford, USA). Zinc oxide nanoparticles, in the form of Nano Tek APS powder with a size range of 40.0–100.0 nm was obtained from Thermo Fisher (Kandel, Germany). The plasma was collected from healthy volunteers and stored at 4 °C in refrigerators for further use.

### Stock and working standard solutions for drugs

#### Standard and working solutions of PTD

In a volumetric flask (50.0 mL), 14.18 mg of PTD was carefully dissolved in an adequate volume of distilled water to produce the PTD in a final stock solution of 1.0 × 10^− 3^ molL^− 1^ and the volume was completed using the same solvent. Serial dilution from the PTD stock solution produced working solutions with concentrations (0.2 ‒ 100.0 µM) and (5.00 -100 nM).

#### Standard and working solutions of PCM

The working solution was prepared from the stock solution of PCM to reach the acquired concentration within the range of 1.0 × 10^− 4^ to 1.0 × 10^− 6^ mol L^− 1^.

### Procedures

#### Modification of SPE with znonps/mwcnt

A suspension of zinc oxide nanoparticles (ZnONPs) and multi-walled carbon nanotubes (MWCNTs) in a 1:1 ratio was prepared in N, N-dimethylformamide (DMF) at a concentration of 1 mg/mL. The mixture underwent ultrasonication for 15 min to achieve uniform dispersion. The homogeneity of the ZnONPs/CNT suspension was verified by optical microscopy, revealing no aggregates larger than 1 μm at 1000× magnification, and further supported by UV–Vis spectroscopy. Subsequently, 10 µL of the homogeneous suspension was drop-cast onto the surface of a screen-printed electrode (SPE). The electrode was then air-dried at room temperature, resulting in the formation of the ZnONPs/CNT-modified SPE (ZnONPs/CNT/MSPE), which was used for subsequent electrochemical analysis.

For comparative studies, individual suspensions of MWCNTs and ZnONPs in DMF were prepared to fabricate MWCNTs/SPE and ZnONPs/SPE, respectively.

All experimental procedures were conducted under stringent safety protocols. Sample preparation involving DMF was performed within certified chemical fume hoods, with all personnel wearing appropriate personal protective equipment (PPE). All waste containing DMF was immediately secured in chemically resistant containers, clearly labeled as “Hazardous Waste– DMF Solution,” and processed through the institution’s hazardous waste management system in accordance with regulatory guidelines. Complete waste manifests were maintained for traceability and compliance.

#### Construction of PTD calibration curve on znonps/cnt/mspe

Creating a calibration plot is a critical step in analytical studies for evaluating quantitative results. Pethidine (PTD) was analyzed using the square wave voltammetry (SWV) technique. Aliquots of PTD within the concentration ranges of 0.2–100 µM and 5.00–100 nM were accurately transferred into 5.0 mL calibrated volumetric flasks using a micropipette and diluted with Britton–Robinson (B–R) buffer at pH 7.0. Square wave voltammograms were recorded using the ZnONPs/CNT-modified screen-printed electrode (ZnONPs/CNT/MSPE). Calibration curves were constructed by plotting the anodic peak current against the corresponding PTD concentrations, thereby establishing a reliable quantitative relationship.

#### Determination of PTD in pharmaceutical formulation

Three *Pethidine*^®^ ampoules (100 mg/2 mL each) were combined, and a 1.0 mL aliquot corresponding to a known amount of PTD was accurately transferred into a 10 mL volumetric flask. The volume was brought to mark using 0.1 M Britton–Robinson (B–R) buffer at pH 7.0. Serial dilutions were then performed to obtain a final PTD concentration of 20.0 × 10^− 3^ mol/L.

Voltammetric measurements were conducted as previously described. For pharmaceutical preparations, PTD concentrations were determined using the standard addition method. Recovery studies were carried out by spiking the sample with standard PTD solutions at concentrations of 4, 30, and 60 µM, each added to a fixed PTD concentration of 20.0 × 10⁻⁶ mol/L.

#### Determination of PTD and PCM in the plasma sample

To prepare the biological sample, 0.5 mL each of paracetamol (PCM) and pethidine (PTD) solutions, both at a concentration of 1.0 × 10^− 3^ mol/L, were mixed with 0.5 mL of untreated human plasma. To precipitate plasma proteins, 3.5 mL of acetonitrile was added to the mixture in a 10 mL centrifuge tube. After centrifugation, 1.0 mL of the resulting supernatant was carefully collected and diluted with deionized water to achieve a final drug concentration of 0.1 mmol/L. A calibration curve was then constructed by plotting the peak current (Ip) against the drug concentration (mol/L), covering a linear range of 5.0 nM to 100 nM.

## Results and discussion

### Investigation of the properties of zno/cnts/mspe

**Fig. 1:**
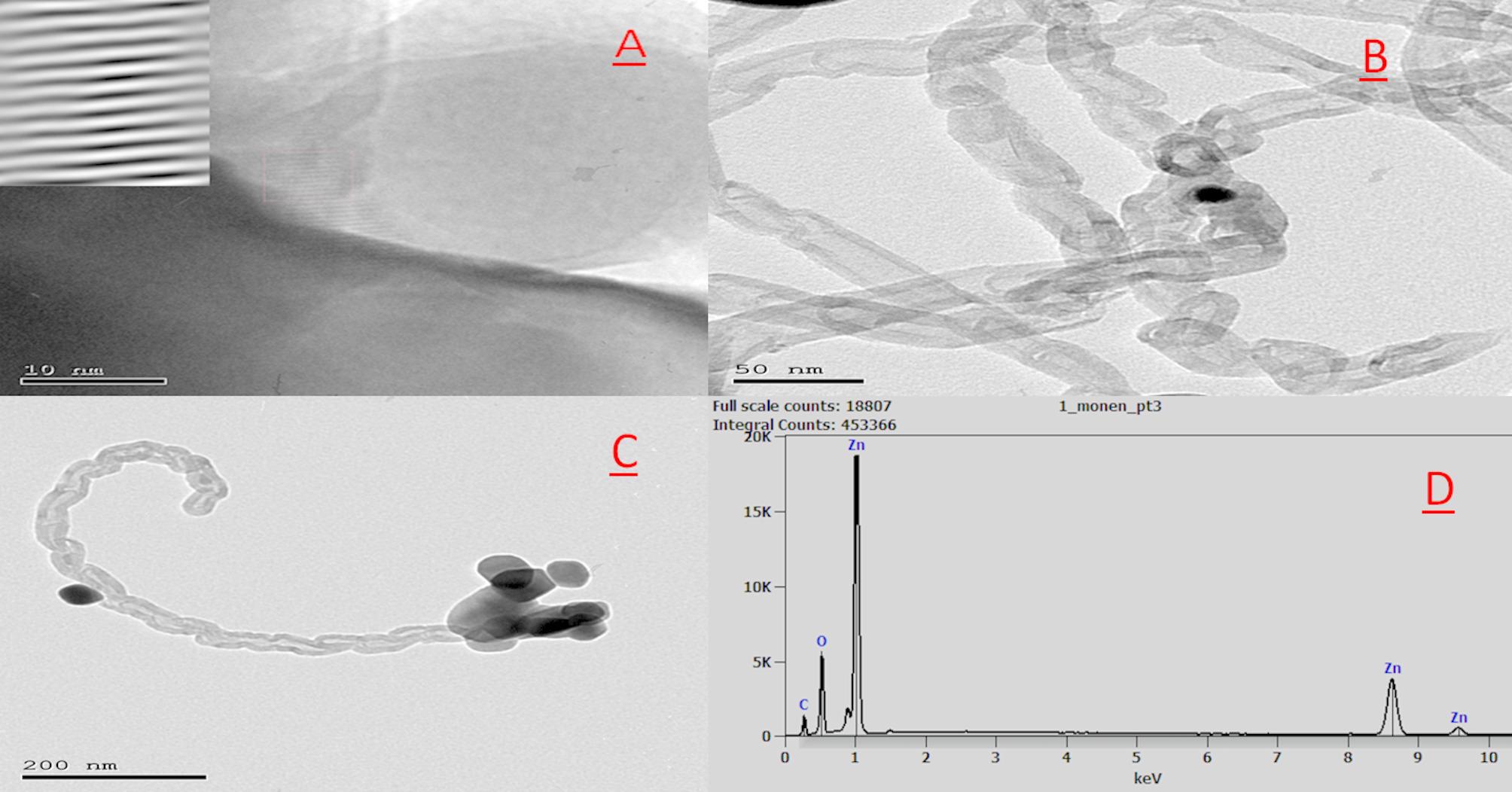
TEM and EDX analysis of the synthesized ZnONPs/MWCNT nanocomposite: (**a**) ZnO nanoparticles (ZnONPs), (**b**) Multi-walled carbon nanotubes (MWCNTs), (**c**) ZnONPs/MWCNT composite showing uniform ZnO distribution on CNT surfaces, (**d**) EDX spectrum confirming elemental composition and weight percentages of the composite

The TEM micrographs and EDX are presented in Fig. 1. In Fig. 1A, the ZnO nanoparticles (ZnONPs) exhibit various morphologies, predominantly near-cubic shapes, which contribute to an increased surface area of the electrode. This enhanced surface area facilitates improved electrochemical sensitivity compared to other ZnO morphologies. Figure 1B shows the TEM image of multi-walled carbon nanotubes (MWCNTs), revealing well-ordered, concentric tubular structures. The outer walls consist of multiple graphitic layers, forming platelet-like structures that are closely aligned with the inner walls of the nanotubes.

Figure 1C illustrates the nanocomposite formed by the integration of ZnONPs with MWCNTs. The ZnO nanoparticles appear to be uniformly distributed across the surfaces of the carbon nanotubes, suggesting a strong interfacial interaction. This uniform dispersion is likely attributed to robust chemical bonding between ZnONPs and the graphitic surfaces of the MWCNTs, contributing to the structural integrity and enhanced performance of the nanocomposite. Figure 1D displays the Energy Dispersive X-ray (EDX) spectrum of the ZnONPs/MWCNT nanocomposite, confirming the elemental composition of the synthesized material. Distinct peaks at approximately 1.0, 8.6, and 9.6 keV correspond to the characteristic X-ray emissions of zinc (Zn), verifying the successful incorporation of ZnO nanoparticles. Additionally, the peaks at around 0.3 keV and 0.5 keV are assigned to carbon (C) and oxygen (O), respectively, indicating the presence of MWCNTs and the oxygen content of ZnO. The pronounced Zn signals, along with the distinct C and O peaks, confirm the formation of the ZnONPs/MWCNT hybrid and demonstrate the effective integration of both components within the nanocomposite matrix.

### Cyclic voltammetric analysis of PTD at znonps/cnt/mspe

Cyclic voltammetry was employed to investigate the voltammetric responses of 5.0 mM K₃Fe(CN)₆ in 0.1 M KCl at both the bare (SPE) and the ZnONPs/CNTs/ (MSPE). These voltammetric results directly illustrate the electrochemistry of PTD on the modified electrode, providing valuable insights into the electrochemical differences between the bare and modified electrodes.

**Fig. 2:**
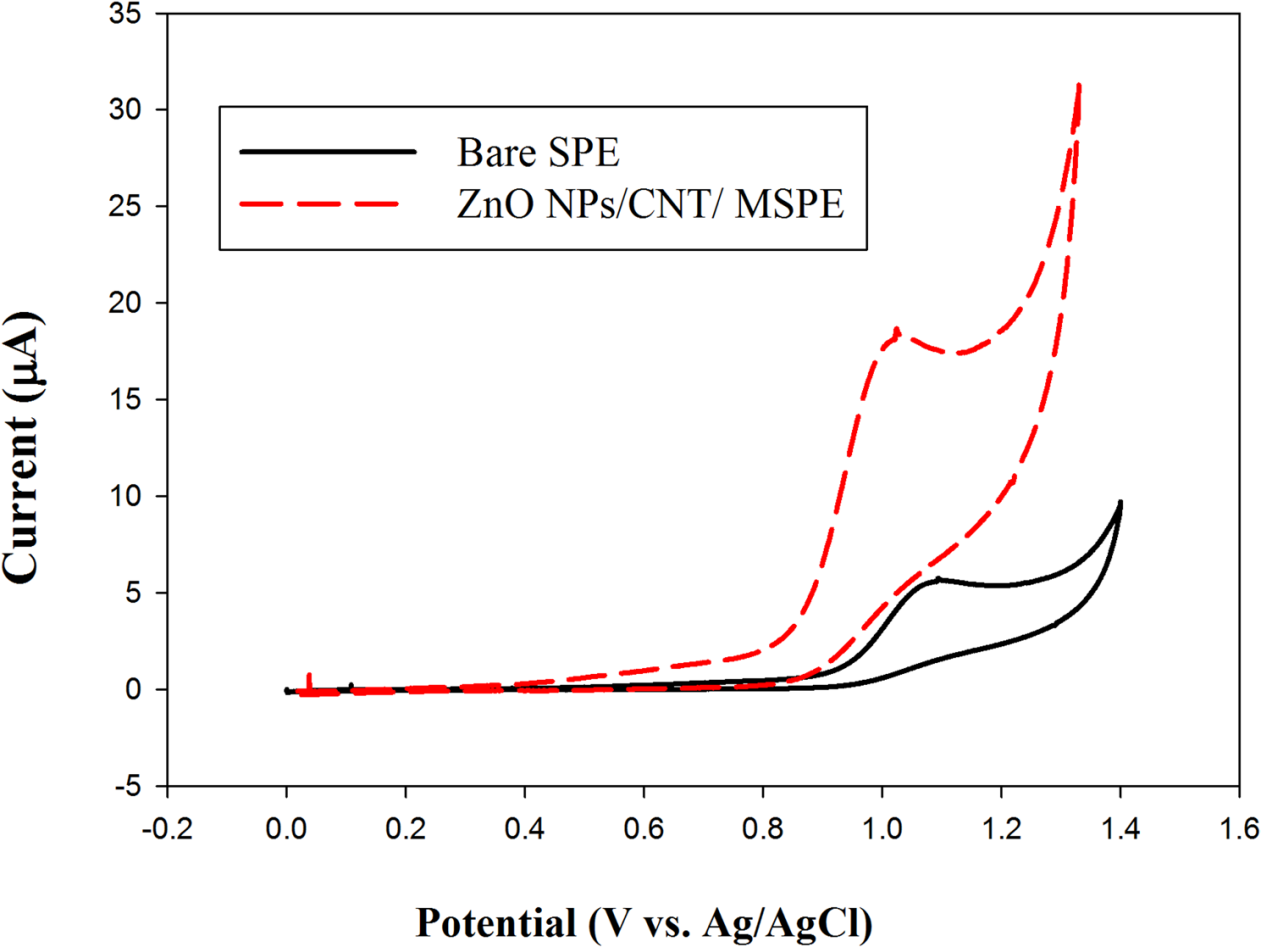
Cyclic voltammograms of 1.0 × 10⁻⁴ M PTD in Britton–Robinson (B–R) buffer (pH 7.0) recorded at a scan rate of 100 mV·s⁻¹ using the bare SPE and the ZnONPs/CNT-modified SPE (ZnONPs/CNT/MSPE)

On the bare SPE surface, the electrochemical oxidation of PTD resulted in a relatively weak and irreversible peak, appearing at a potential of 1.09 V. In contrast, as shown in Fig. 2, the incorporation of ZnO/CNTs/MSPE significantly enhanced the anodic current to 19 µA. This distinct increase in the anodic peak current on the ZnO/CNTs/MSPE surface, compared to the bare SPE, indicates the superior electrochemical performance of the modified electrode.

Furthermore, the oxidation potential at the ZnO/CNTs/MSPE was negatively shifted from 1.09 V to 1.002 V. This potential shift suggests that the CNTs may exert a catalytic effect on the oxidation of PTD.

The exceptional sensitivity of PTD detection at the ZnONPs/CNT/MSPE can be attributed to several synergistic factors: (1) the cation-exchange capability of ZnO, which enhances electrostatic interactions with the analyte; (2) the inherent electrocatalytic activity of CNTs, previously associated with residual metal impurities and the presence of edge-plane-like defects at the nanotube ends [35, 36]; and (3) the increased surface porosity of the modified electrode, which facilitates improved analyte accessibility and electron transfer, thereby enhancing overall electrochemical performance.

### Adjustment of the experimental factors

Several experimental parameters were optimized to establish the ideal conditions for PTD analysis in pharmaceutical formulations, pure drug samples, human plasma, and in the presence of co-administered paracetamol (PCM). This study specifically examined the influence of solution pH and scan rate on the electrochemical response.

#### Influence of pH

**Fig. 3:**
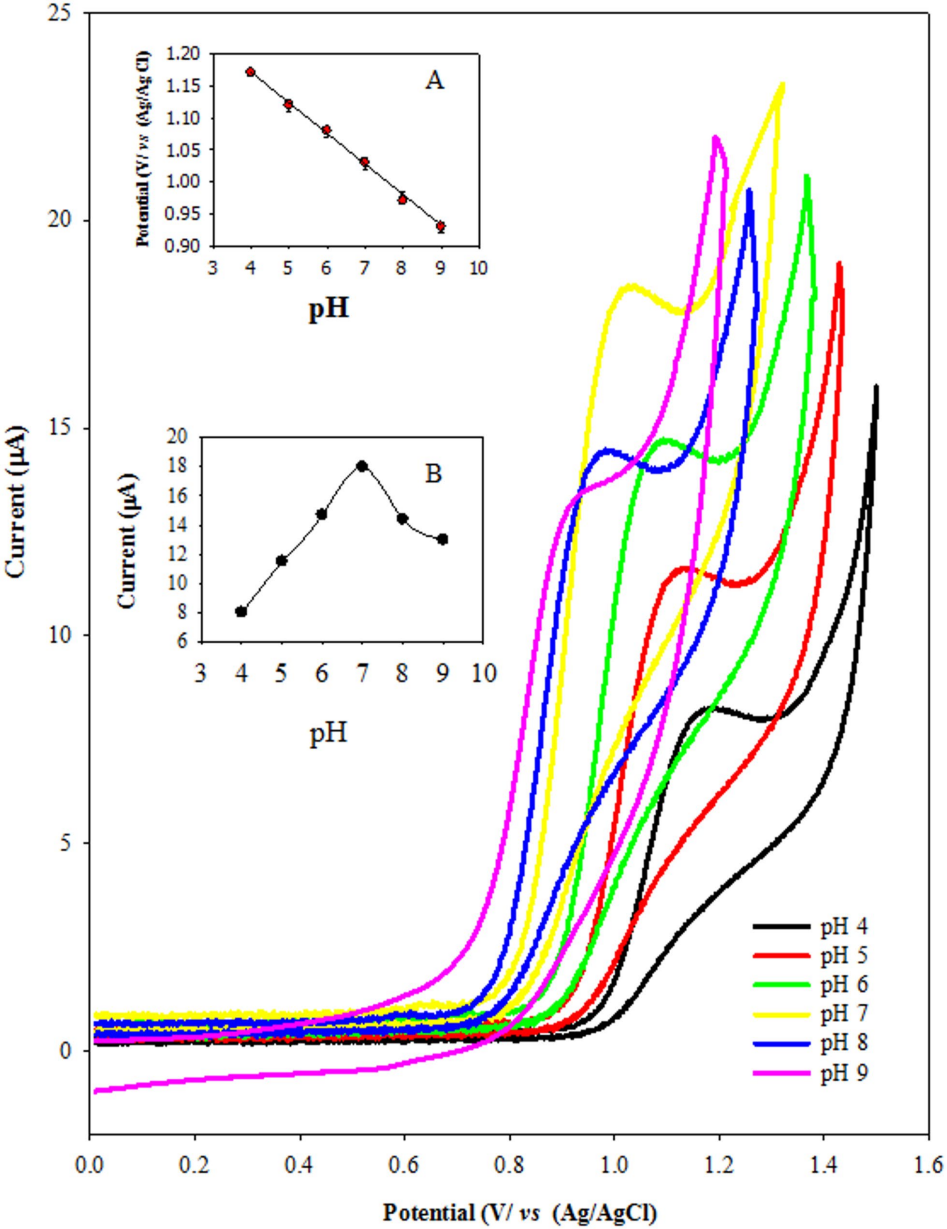
Cyclic voltammetric responses of 1.0 × 10^− 4^ M of PTD at different pH values using ZnONPs/CNT/MSPE sensing platform at Scan rate 100 mV s^− 1^. (**A**) shows a plot of anodic peak potential of PTD versus pH at the modified electrode (**B**) shows a plot of anodic peak current of PTD versus pH at modified electrode

The electrochemical behavior of PTD, including its sensitivity and voltammetric peak resolution, is significantly influenced by the pH of the supporting electrolyte. As illustrated in Fig. 3A, cyclic voltammograms of PTD were recorded in Britton–Robinson (B–R) buffer over a pH range of 4.0 to 9.0. A progressive negative shift in the oxidation peak potential was observed with increasing pH, indicating a proton-dependent oxidation process. Figure 3B demonstrates a strong linear correlation between the oxidation peak potential (Ep) of PTD and pH, described by the equation:

1 $$\text{P}\text{T}\text{D}:\:\text{E}\text{p}\:\left(\text{V}\right)=\:1.3618\:-\:0.0477\:\text{p}\text{H}({\text{R}}^{2}\:=\:0.9995)$$

The slope of − 0.0477 V/pH unit closely approximates the theoretical Nernstian slope of − 59 mV per pH unit at 25 °C, which is consistent with an electrochemical oxidation mechanism involving the transfer of an equal number of protons and electrons. The maximum peak current was observed at pH 7.0, near the pKa of PTD (8.6) [37], suggesting favorable protonation conditions for optimal electron transfer. Therefore, pH 7.0 was selected as the optimal condition for all subsequent experiments. These findings confirm that the oxidation of PTD is a pH-dependent process, in accordance with the Nernst equation.

#### Electrochemical response of PTD at varying scan rates

**Fig. 4:**
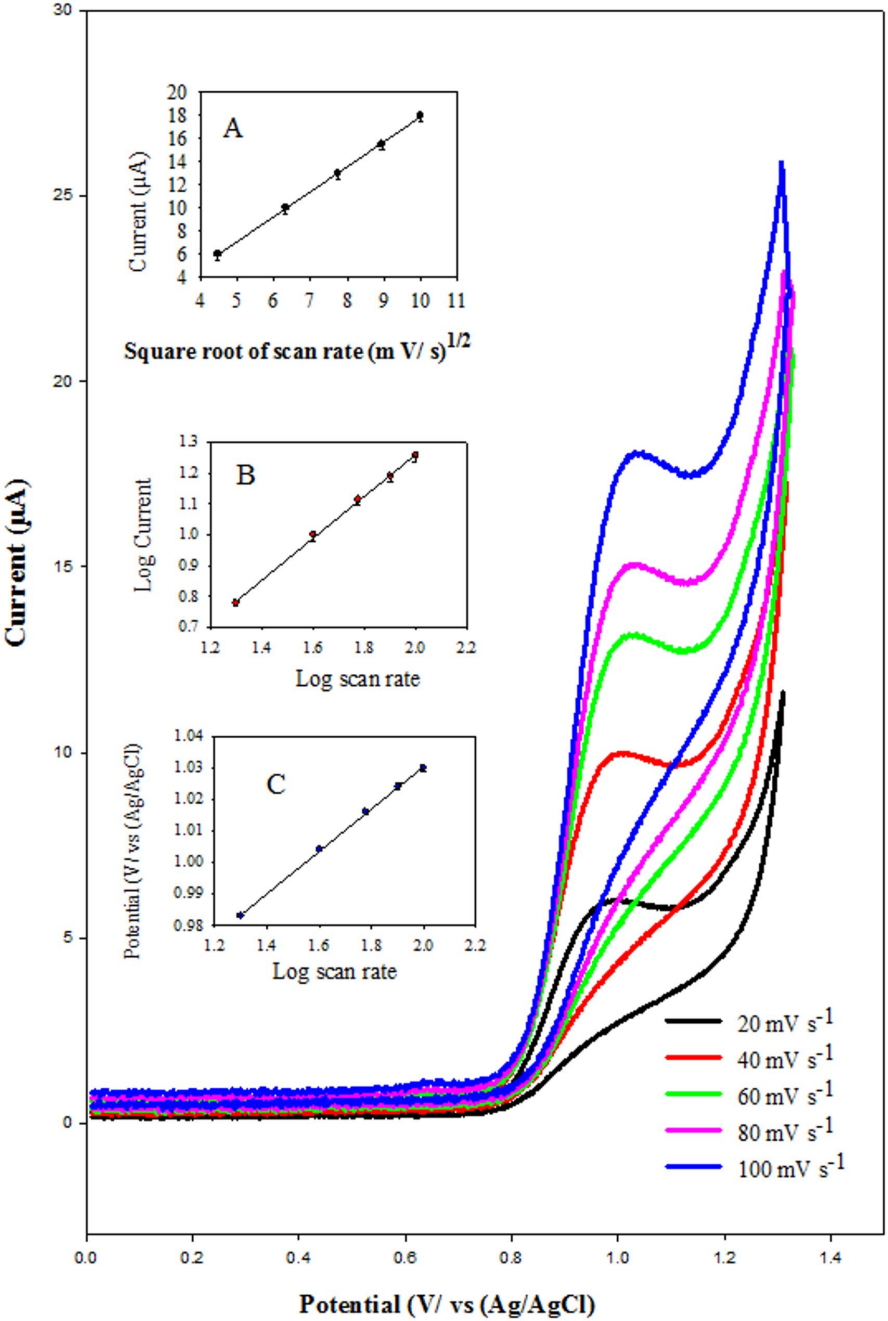
Cyclic voltammetric responses of 1.0 × 10^− 4^ M PTD in Britton–Robinson buffer (pH 7.0) recorded at various scan rates (20.0–100.0 mV s^− 1^) using the ZnONPs/CNT-modified screen-printed electrode (ZnONPs/CNT/MSPE): (**A**) illustrates the linear relationship between the anodic peak current and the square root of the scan rate (ν^¹/²^), (**B**) shows the log–log plot of anodic peak current versus scan rate, (**C**) displays the linear relationship between peak potential (Ep) and the logarithm of scan rate

The electrochemical behavior of 0.1 mmol·L⁻^1^ pethidine (PTD) in Britton–Robinson (B–R) buffer at pH 7.0 was evaluated across a range of scan rates (20–100 mV s⁻^1^) to elucidate the charge transfer mechanism—whether governed by diffusion, adsorption, or a combination of both. As shown in Fig. 4A, the peak current (Ip) exhibited a linear correlation with the square root of the scan rate (ν^¹/²^), consistent with diffusion-controlled kinetics, and in accordance with the Randles–Ševčík equation [38]:

2 $$\text{I}\text{p}=\left(2.15\:\times\:\:{10}^{5}\right){\text{n}}^{3/2}\:\text{A}\:{\text{C}}_{0}\:{\text{D}}_{0}^{1/2}\:{{\nu\:}}^{1/2}$$

Where *Ip* is the peak current (µA), *n* is the number of electrons involved in the redox process, A is the effective electrode surface area (0.0948 cm² for ZnONPs/CNT/MSPE), *C₀* is the analyte concentration, *D₀* is the diffusion coefficient (cm²s⁻^1^), and *ν* is the scan rate (V s⁻^1^).

Using square wave voltammetry (SWV), the apparent diffusion coefficient (Dapp) of PTD was calculated. It increased significantly from 9.01 × 10⁻⁷ cm² s⁻^1^ for the bare SPE to 5.93 × 10⁻^5^ cm² s⁻^1^ for the ZnONPs/CNT-modified SPE, indicating a substantial enhancement in mass transport and electron transfer efficiency at the modified surface [39]. In Fig. 4B, a log–log plot of peak current (Ip) versus scan rate (ν) yielded the regression equation:

3 $$\text{l}\text{o}\text{g}\:\text{I}\text{p}\:=\:0.6792\:+\:0.0986\:\text{l}\text{o}\text{g}\:{\nu\:}\:({\text{R}}^{2}\:=\:0.9992)$$

The slope (~ 0.5) suggests that the electrochemical oxidation of PTD is primarily governed by diffusion. Among all scan rates tested, 100 mV s⁻^1^ provided the most distinct and sensitive voltammetric response. As shown in Fig. 4C, the oxidation peak potential (Ep) shifted positively with increasing scan rate, indicative of an irreversible electron-transfer process. The relationship is given by:

4 $$\text{E}\text{p}\:\left(\text{V}\right)=\:0.8956\:+\:0.0675\:\text{l}\text{o}\text{g}\:{\nu\:}({\text{R}}^{2}\:=\:0.9997)$$

To further investigate the electron-transfer kinetics, Laviron’s equation for irreversible systems was applied [40]:

5 $$\begin{array}{l}\text{E}\:=\:{\text{E}}^{0}\:+\:(2.303\text{R}\text{T}\:/\:{\alpha\:}\text{n}\text{F})\:\times\:\:\text{l}\text{o}\text{g}({\text{R}\text{T}\text{k}}^{0}\:/\:{\alpha\:}\text{n}\text{F})\:\\+\:(2.303\text{R}\text{T}\:/\:{\alpha\:}\text{n}\text{F})\:\times\:\:\text{l}\text{o}\text{g}\:{\nu\:}\end{array}$$

Where *E* is the peak potential, *R* is the universal gas constant (8.314 J mol⁻^1^ K⁻^1^), *T* is the temperature (298 K), *F* is the Faraday constant (96,485 C mol⁻¹), *α* is the electron transfer coefficient, *n* is the number of electrons transferred, and *ν* is the scan rate. The slope of the Ep vs. log ν plot (0.08956) allowed for the calculation of the product αn = 0.661. Assuming α ≈ 0.5 (a typical value for irreversible systems), the number of electrons (*n*) involved in the oxidation process was estimated to be 1.321, confirming that one electron participates in the electro-oxidation of PTD at the ZnONPs/CNT/MSPE.

### Oxidation mechanism of PTD

Based on the above-mentioned data, the electro-oxidation of PTD can be characterized as an irreversible, diffusion-controlled process involving the transfer of one electron and one proton. The voltammetric behavior of PTD at the modified electrode suggests an electron transfer mechanism leading to the formation of a radical cation, most likely localized at the nitrogen atom. The electrochemical response of PTD at the ZnONPs/CNT-modified screen-printed electrode (SPE) indicates that the oxidation process is influenced by surface interactions, with the electron transfer rate governed by the affinity of PTD molecules toward the modified electrode interface [19]. Schematic 5 provides a mechanistic representation of the proposed electro-oxidation pathway of PTD at the ZnONPs/CNT/MSPE surface.

**Fig. 5:**
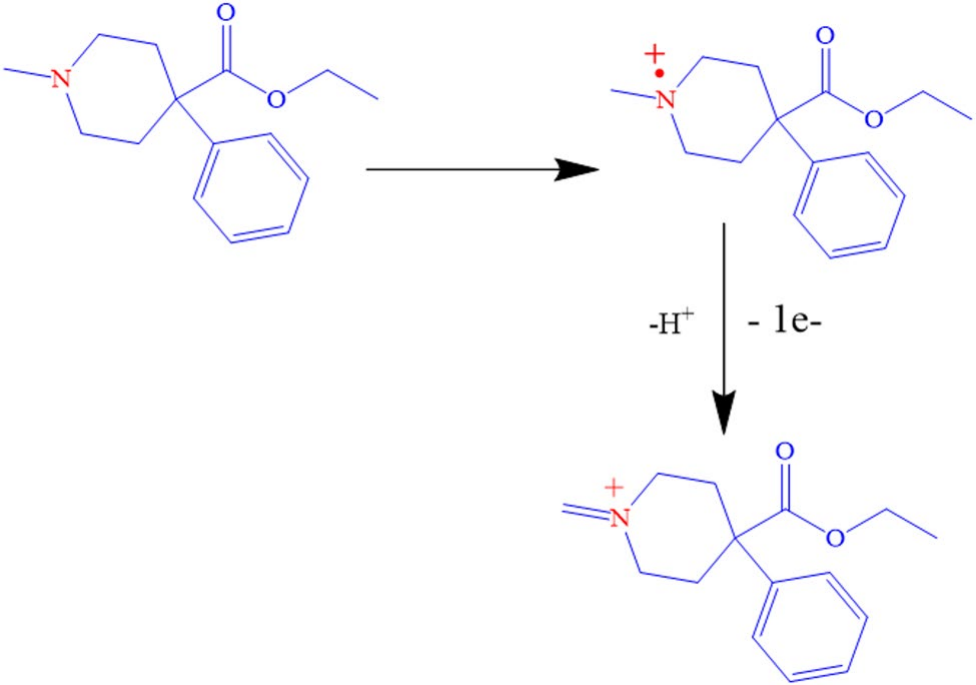
Electrochemical oxidation reaction of PTD.

## Method validation

In accordance with the International Conference on Harmonization (ICH) [41] guidelines, Table 1 displays the findings of the calculation of several verification parameters, including linearity, precision, accuracy, LOQ, LOD, specificity, and robustness.

**Table 1:** Validation parameters of the proposed voltammetric method for the determination of PTD using modified ZnONPs/CNT/MSPE

Parameters	1st Linear segment	2nd linear segment
**Linearity**	5.00–100.0 (nM)	0.20–100.0(µM)
**LOD (pM)**	183	
**LOQ (pM)**	554	
**Slope (µA/M)**	0.0620	0.0903
**Intercept (µA)**	0.8357	8.0706
**Correlation coefficient (r)**	0.9992	0.9997
**Accuracy Mean ± SD**	98.24 ± 2.173	99.63 ± 1.805
**Precision (%RSD)**		
**Repeatability**	1.031	0.946
**Intermediate precision**	2.042	1.794

### Linearity

**Fig. 6:**
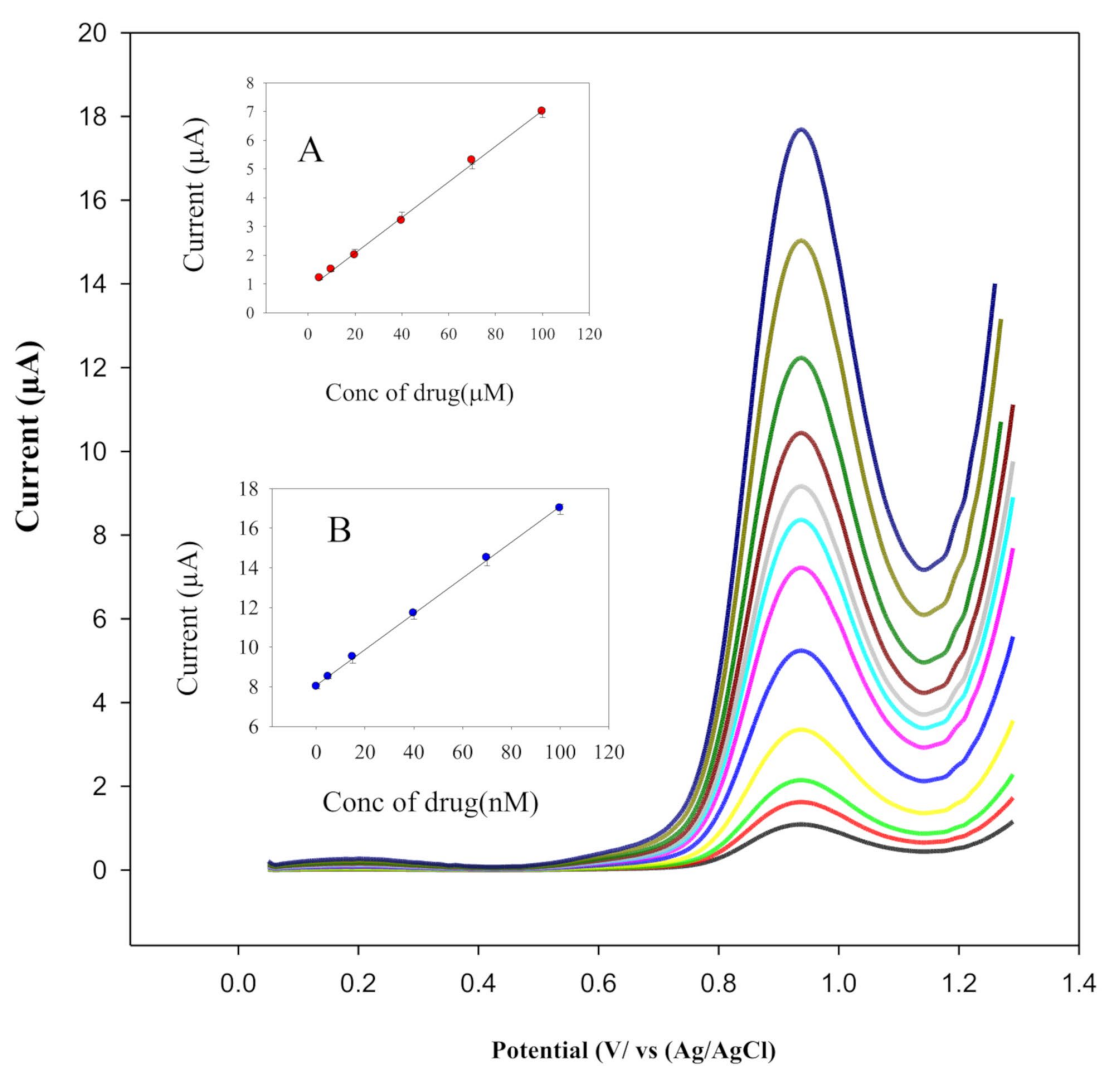
Square wave voltammograms of PTD at various concentrations in B-R buffer pH 7.0, recorded at a scan rate of 20 mVs^–1^ using ZnONPs/CNT/MSPE. Inset Fig. 6**A** displays the calibration curve for PTD over the concentration range of 0.2–700.0 nM, while Fig. 6**B** shows the calibration curve for the range of 5.00–100.0 µM

To evaluate the effectiveness of the proposed method for quantitative determination of PTD under the optimized conditions, square wave voltammetry (SWV) was employed to assess the linearity of the sensor response (Fig. 6). A clear linear correlation was observed between the anodic peak current and PTD concentration. As shown in Fig. 6A, the sensor demonstrated high sensitivity, enabling the detection of PTD in the low nanomolar range (0.2–700.0 nM). Figure 6B further confirms the sensor’s ability to accurately quantify PTD over a broader concentration range (5.00–100.0 µM), with strong correlation coefficients in both cases. The corresponding analytical parameters, including regression equations, linearity ranges, and limits of detection, are summarized in Table 1.

### Limit of detection and quantification

The limit of detection (LOD) and limit of quantification (LOQ) for PTD were calculated based on the standard formulas: LOD = 3.3 σ/S and LOQ = 10 σ/S, where σ is the standard deviation of the intercept and S is the slope of the calibration curve. The resulting values were 183 pmol L^**–1**^ for LOD and 554 pmolL^**–1**^ for LOQ. These results confirm the high sensitivity of the ZnONPs/CNT/MSPE sensor, supporting its suitability for trace-level PTD analysis, as summarized in Table 1.

### Accuracy and precision

The precision of the developed method was rigorously assessed by analyzing three distinct concentrations of PTD. Repeatability was evaluated through multiple measurements conducted on the same day, while intermediate precision was determined over three consecutive days. The relative standard deviations (RSDs) for all measurements were consistently below 2%, indicating excellent reproducibility. These results, summarized in Table 1, confirm the high reliability and precision of the proposed method.

### Robustness

The procedure’s reliance is confirmed by the reproducibility of the peak current with mild alterations in experimental circumstances. The pH (7.0 ± 0.2) and measurement duration (20s ± 4s) were tested. The study medication’s maximum peak current intensity was unaffected, demonstrating the effectiveness of the recommended system under typical use, less than 2% were observed in Table 1.

### Specificity and selectivity

The method’s ability to determine the PTD in the medication without being affected by excipients or often present organic and inorganic compounds has demonstrated its specificity.

### Quantitative determination of pethidine (PTD) in pharmaceutical formulations

The square wave voltammetry (SWV) technique was successfully applied for the quantification of pethidine (PTD) in a pharmaceutical formulation, as presented in Table 2. The method’s applicability was further validated using the standard addition method. The obtained results were statistically compared with those of the official method [5]. As shown in Table 3, no significant differences in accuracy or precision were observed between the proposed and official methods. The calculated *t*- and *F*-values were within the 95% confidence limits and below the critical values, confirming the validity and reliability of the proposed method for PTD determination.

**Table 2:** Determination of PTD in a pharmaceutical formulation using znonps/cnt/ MSPE

Sample	Claimed conc.	Amount added standard PTD (µM)	Apparent recovery% PTD
**Pethidine Injection 100 mg/2ml**	20.0 × 10^− 6^	4.00	98.52
		30.00	99.07
		60.00	99.81
**Recovery%±RSD**	99.13 ± 0.647		

**Table 3:** Statistical comparison between the results of the determination of pure PTD by the proposed voltametric method and the official method

Parameters	Proposed voltammetric method for PTD ZnONPs/ CNT/ MSPE	Official method ^a^
**Mean**	99.37	99.05
**SD**	2.196	1.541
**n**	5	5
**Variance**	4.392	3.082
***t*** **(2.228)** ^**b**^	0.283	
**F (5.05)** ^**b**^	2.030	

^**a**^ HPLC method using C_18_ column and a mixture of acetonitrile: phosphate buffer as mobile phase. The UV detection was performed at 257.0 nm using a UV detector

^**b**^ The values in parentheses are the corresponding tabulated values at *p*

### Simultaneous determination of PTD and Co-administered drug PMC

**Fig. 7:**
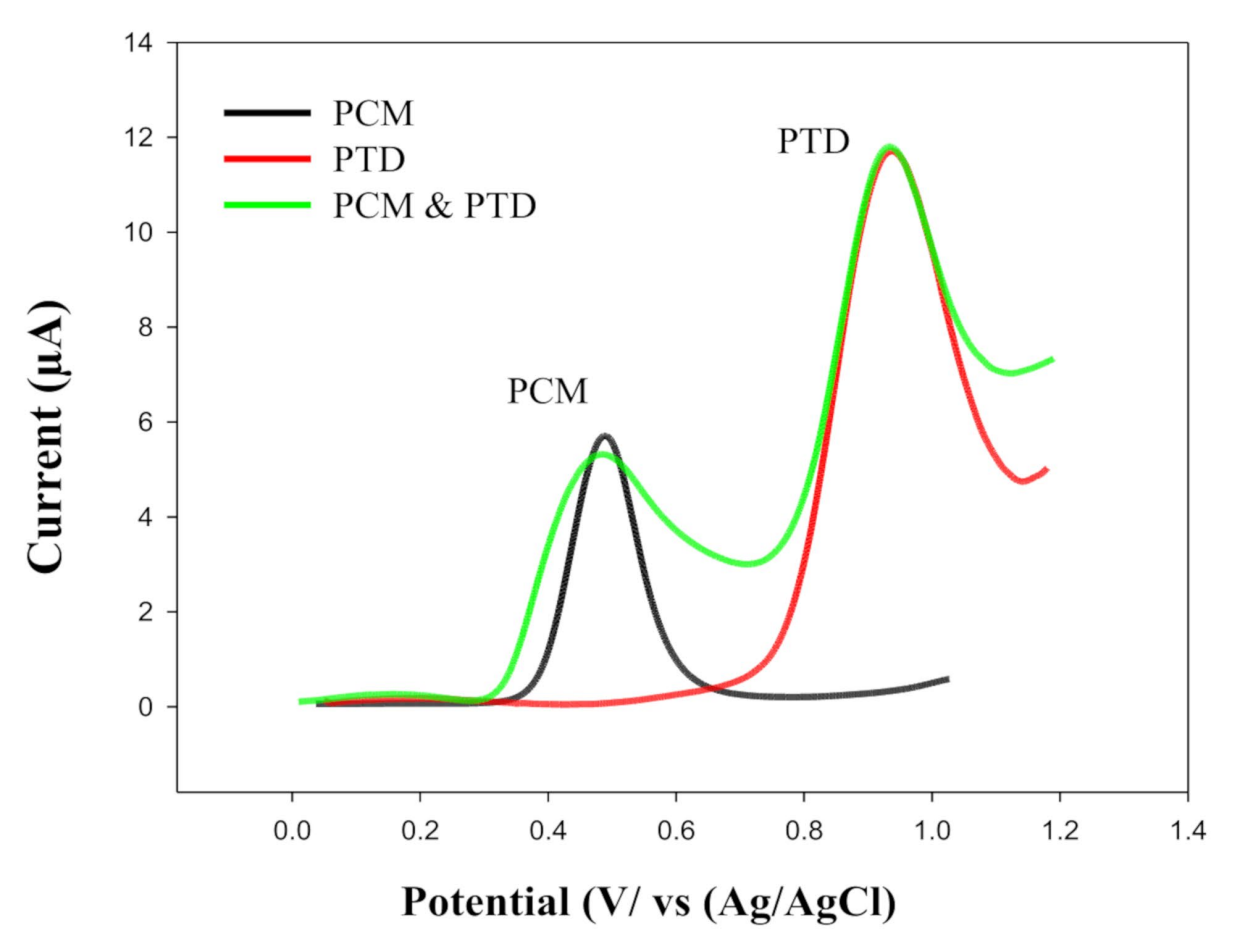
Square wave voltammograms of 10 µM PTD, PCM, and their mixture recorded using the ZnONPs/CNT-modified screen-printed electrode in Britton–Robinson buffer (pH 7.0) at a scan rate of 20 mV s^− 1^

Paracetamol (PCM) is often co-administered with pethidine (PTD) for the reasons outlined in the introduction, making the simultaneous determination of both drugs clinically important. To enable their electrochemical detection, the voltammetric behavior of PCM and PTD was individually studied using a ZnONPs/CNT-modified screen-printed electrode (ZnONPs/CNT/MSPE). A mixture of both drugs was also analyzed at the same electrode, as shown in Fig. 7. The figure displays well-separated oxidation peaks at 0.473 V for PCM and 0.930 V for PTD, with a potential difference (ΔE) of 0.457 V (457 mV). This excellent electrochemical resolution confirms the feasibility of simultaneous detection and highlights the strong electron mediation capability of the modified sensor.

**Fig. 8:**
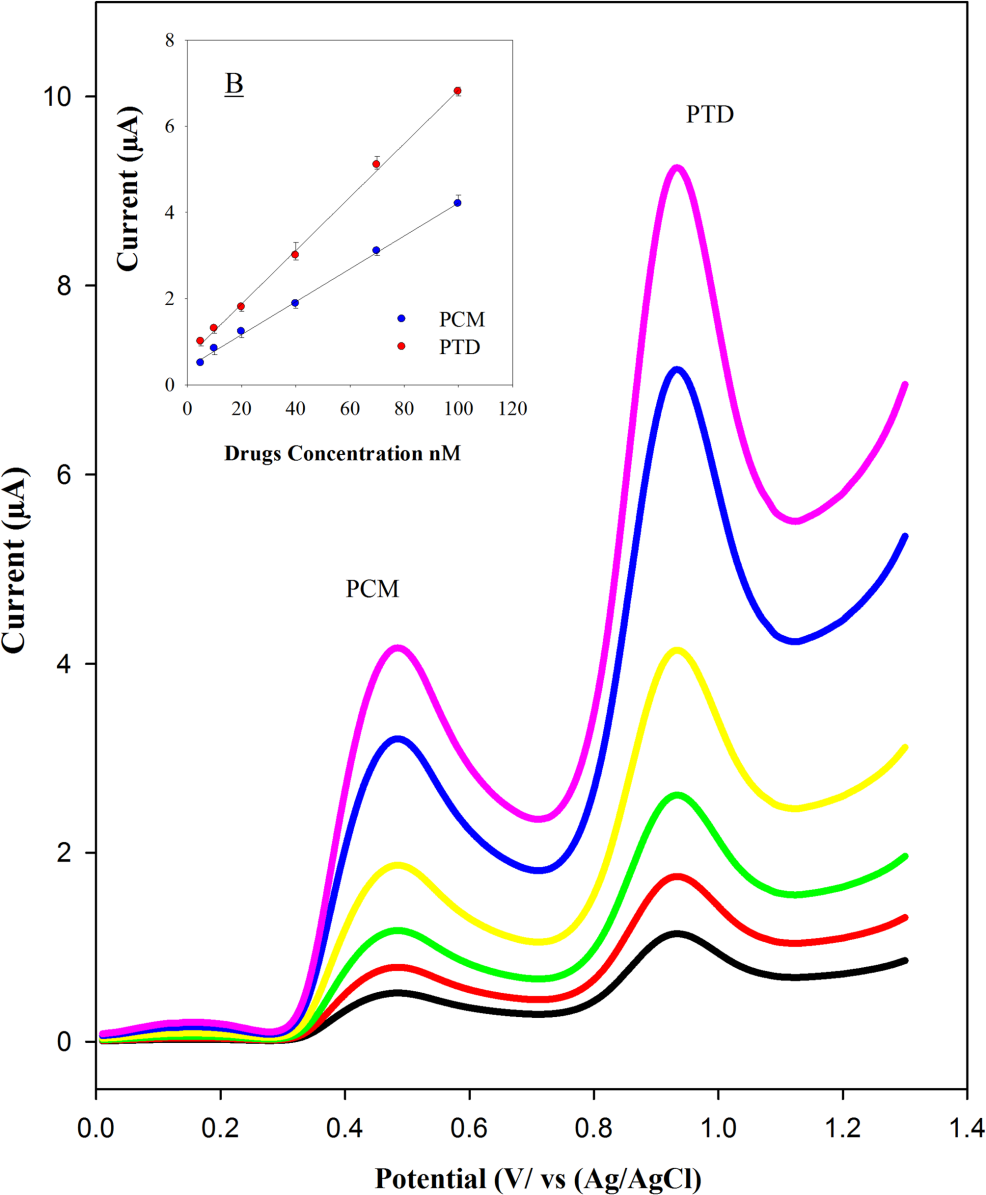
Square wave voltammograms were recorded for solutions containing varying concentrations of PCM and PTD (5–100 nM) using the ZnONPs/CNT-modified screen-printed electrode in Britton–Robinson buffer (pH 7.0) at scan rate 20 mV^− 1^. The inset illustrates the corresponding calibration curves for the quantitative determination of both PCM and PTD

Simultaneous determination of the PTD and PCM mixture in plasma was performed, as shown in Fig. 8A, which presents square wave voltammograms (SWVs) for solutions containing varying concentrations of PTD and PCM (5.0–100 nM). A clear increase in peak currents was observed with rising PCM concentrations. Figure 8B shows a strong linear correlation between peak currents and the concentrations of both analytes, confirming the method’s sensitivity and reliability.

### Effect of interfering materials

To assess potential interferences in the electrocatalytic detection of PTD and PCM, we examined the effects of various commonly encountered substances, including dopamine, ascorbic acid, uric acid, glucose, starch, sucrose, cellulose, and physiologically relevant ions (Na⁺, K⁺, Mg²⁺). The electrochemical responses of PTD and PCM remained stable in the presence of these compounds, indicating the high selectivity of the developed sensor. These findings confirm that the proposed electroanalytical method is not affected by common interferences, ensuring accurate and reliable quantification of PTD and PCM in complex biological matrices, as presented in Table 4.

**Table 4:** The effect of interference material on the electro-analytical determination of PTD and PCM by SWVs

Interference material 0.1 mmoL^− 1^	RSD % of peak current PTD	RSD % of peak current PCM
**Dopamine**	2.01	1.97
**Uric acid**	2.11	1.95
**Ascorbic acid**	1.90	2.04
**Starch**	1.86	1.89
**Sucrose**	2.41	2.08
**Glucose**	1.94	1.79
**Mg**,** K**,** Na ions**	2.38	2.50
**Cellulose**	1.99	2.16

## Conclusion

This study presents the first electrochemical method for the simultaneous detection of pethidine (PTD) in the presence of the co-administered drug paracetamol (PCM). A screen-printed electrode (SPE) modified with zinc oxide nanoparticles and multi-walled carbon nanotubes (ZnO NPs/MWCNTs) was developed to enhance electron transfer and improve sensitivity. The sensor enabled accurate and reliable determination of PTD in both pure form and pharmaceutical formulations, as well as in plasma samples containing PCM. The proposed electroanalytical approach is simple, effective, and unique compared to previously reported methods, offering direct detection without the need for complex sample preparation. Additionally, the ZnO NPs/MWCNTs-modified SPE is cost-effective, easy to fabricate, and user-friendly, making it a promising tool for routine drug analysis and point-of-care diagnostics. With further development, this sensor could find significant applications in both clinical and forensic settings.

## Supplementary Information

Below is the link to the electronic supplementary material.

Supplementary Material 1

## Data Availability

Availability of data and materialsDatasets generated and/or analyzed during the current study are available from the corresponding author on reasonable request.
