# Supplementary figures and images for: Advanced screen-printed electrode functionalized ZnO/CNTs for the electrochemical analysis of opioid drug pethidine co administered with paracetamol: application in dosage form and human plasma sample
Source: BMC Chem. 2025 Aug 9;19(1):235. doi: 10.1186/s13065-025-01599-8 (PMC12335068; doi:10.1186/s13065-025-01599-8)

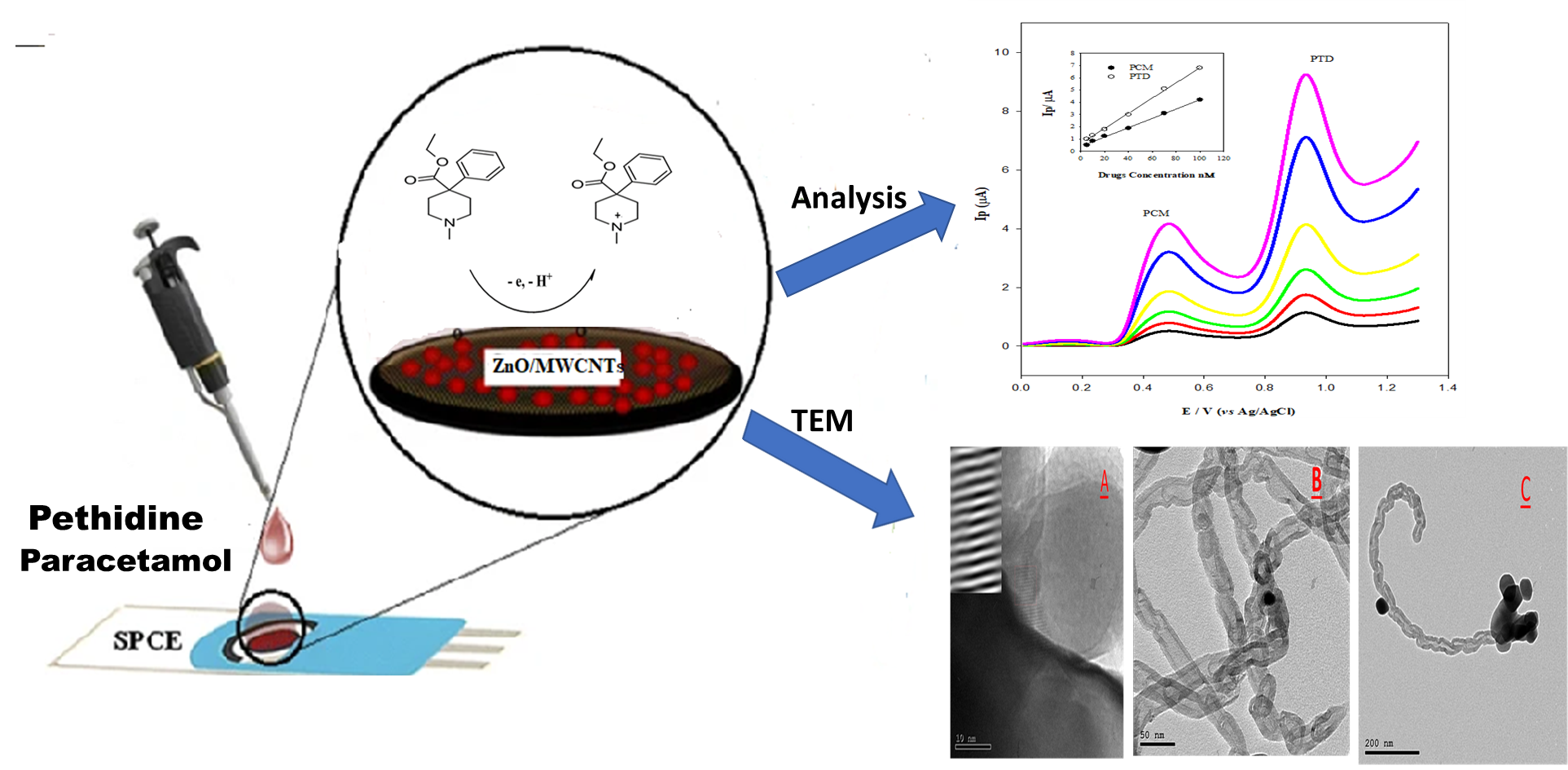

Supplement: Supplementary file 1 — Supplementary Material 1 [file 13065_2025_1599_MOESM1_ESM.doc]
